# DNA methylation modulated genetic variant effect on gene transcriptional regulation

**DOI:** 10.1186/s13059-023-03130-5

**Published:** 2023-12-08

**Authors:** Yong Zeng, Rahi Jain, Magnus Lam, Musaddeque Ahmed, Haiyang Guo, Wenjie Xu, Yuan Zhong, Gong-Hong Wei, Wei Xu, Housheng Hansen He

**Affiliations:** 1 Princess Margaret Cancer Centre, University Health Network, Toronto, Canada; 2 Department of Medical Biophysics, University of Toronto, Toronto, Canada; 3 Department of Clinical Laboratory, the Second Hospital, Cheeloo College of Medicine, Shandong University, Jinan, 250033 Shandong China; 4 MOE Key Laboratory of Metabolism and Molecular Medicine and Department of Biochemistry and Molecular Biology of School of Basic Medical Sciences, Fudan University Shanghai Cancer Center, Shanghai Medical College of Fudan University, Shanghai, China; 5 Biocenter Oulu & Faculty of Biochemistry and Molecular Medicine, University of Oulu, Oulu, Finland; 6 Dalla Lana School of Public Health, University of Toronto, Toronto, ON Canada

**Keywords:** SNP, CTCF, meCpG, eQTL, Memo-eQTL, Chromatin 3D structure

## Abstract

**Background:**

Expression quantitative trait locus (eQTL) analysis has emerged as an important tool in elucidating the link between genetic variants and gene expression, thereby bridging the gap between risk SNPs and associated diseases. We recently identified and validated a specific case where the methylation of a CpG site influences the relationship between the genetic variant and gene expression.

**Results:**

Here, to systematically evaluate this regulatory mechanism, we develop an extended eQTL mapping method, termed DNA methylation modulated eQTL (memo-eQTL). Applying this memo-eQTL mapping method to 128 normal prostate samples enables identification of 1063 memo-eQTLs, the majority of which are not recognized as conventional eQTLs in the same cohort. We observe that the methylation of the memo-eQTL CpG sites can either enhance or insulate the interaction between SNP and gene expression by altering CTCF-based chromatin 3D structure.

**Conclusions:**

This study demonstrates the prevalence of memo-eQTLs paving the way to identify novel causal genes for traits or diseases associated with genetic variations.

**Supplementary Information:**

The online version contains supplementary material available at 10.1186/s13059-023-03130-5.

## Background

Genome-wide association studies (GWAS) have identified over 300,000 SNP-trait associations [1]. However, the vast majority (> 90%) of these disease-associated risk single-nucleotide polymorphisms (rSNPs) are located in non-coding regions, complicating their functional evaluation [2]. Expression quantitative trait locus (eQTL) mapping is a valuable tool to elucidate the relationship between genetic variants and gene expression, helping to bridge the gap between rSNPs and associated diseases by identifying potential causal genes [3, 4]. There is a more significant overlap between rSNPs and eQTLs than one would expect by chance alone (as reviewed in [5]), and the overlapped SNPs are often enriched in cis-regulatory elements (CREs) influencing transcriptional regulation [6]. Despite this, a large number of rSNPs remain untagged by any target genes through eQTL analysis.

In a recent study, we identified prostate cancer rSNP, rs11986220, as a novel eQTL for the oncogenic gene MYC in a subset of samples with a high level of methylation at a CpG site located approximately 10 kilobase pairs (kbp) upstream of the MYC promoter. We demonstrated that high DNA methylation at this site prevents CTCF binding and the formation of a chromatin loop, which would allow for a long-range interaction between this rSNP and MYC [7]. Unlike SNPs that are located in CTCF binding sites and directly affect high-order chromatin architecture [8, 9], this rSNP is located in an enhancer region about 210kbp away from the CTCF binding site [7]. This study highlights a functional mechanism in which DNA methylation acts as a moderator to regulate the relationship between genetic variant and gene expression by affecting CTCF-based 3D chromatin architecture.

To systematically evaluate the DNA methylation modulated relationship between genetic variant and gene expression, we introduced a novel eQTL analysis method named DNA methylation modulated eQTL (memo-eQTL). The modulation effect was statistically characterized as the interaction between the SNP and methylated CpG site (SNP × meCpG), and its significance was determined by comparing the multiple regression models with and without the interaction [10, 11]. Analysis of 128 normal prostate tissue samples led to the identification of 1063 memo-eQTLs, marking them as a novel type of eQTLs. This method holds promise in identifying a novel category of eQTLs influenced by DNA methylation modulation.

## Results

### Identification of meCpG sites associated with CTCF occupancy

Our previous study demonstrated that a meCpG can modulate the interaction between genetic variant and gene expression by altering CTCF binding [7]. Building on this, we hypothesized that other meCpGs sites, where methylation levels are associated with CTCF occupancy, might exert similar effects. To test this hypothesis, we analyzed the correlation of 444,364 meCpG-CTCF pairs across 26 human cell lines or tissues, including the prostate gland, using matched whole-genome bisulfite sequencing (WGBS) and CTCF chromatin immunoprecipitation sequencing (ChIP-seq) data from ENCODE (Additional file 5: Fig. S1A, B) [12]. Of these, 10,987 meCpG-CTCF pairs showed significant negative correlation, while only 246 pairs displayed a positive correlation (Additional file 5: Fig. S1C and Additional file 1: Table S1).

**Fig. 1:**
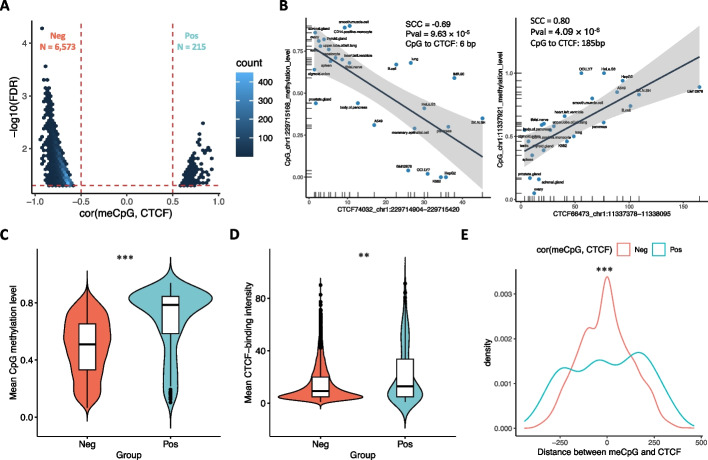
Correlation between meCpG and CTCF binding. **A** The correlation coefficients and statistical significance for the most significantly correlated meCpG and CTCF per each CTCF binding site. Neg and Pos refer to negatively correlated and positively correlated meCpG-CTCF pairs, respectively. **B** Examples of negatively and positively correlated meCpG-CTCF pair across 26 ENCODE samples (SCC: Spearman correlation coefficient; Pval: *p*-value; CpG to CTCF: the distance from the meCpG site to the center of the CTCF binding site). Comparisons of the average CpG methylation levels (**C**) and average CTCF binding intensity (**D**) between Neg and Pos groups (Wilcoxon rank-sum two-sided test: mean CpG methylation level: *p* < 2.20 × 10^−16^; mean CTCF intensity: *p* = 2.20 × 10^−3^). **E** Comparison of the distances between the meCpG site and the center of CTCF binding site for Neg and Pos groups (Kolmogorov–Smirnov test: *p* = 1.05 × 10.^−5^). **p < 0.01; ***p < 0.001

Although a single CTCF binding site might contain multiple meCpG sites (Additional file 5: Fig. S1D), we only identified meCpG-CTCF pairs showing opposite correlations in five CTCF binding sites, suggesting that meCpG sites located in the same CTCF binding site likely share a similar relationship with CTCF occupancy. Thus, after removing these five CTCF binding sites, we selected the most significantly correlated meCpG-CTCF pair for each CTCF binding site, resulting in 6573 negatively correlated and 215 positively correlated meCpG-CTCF pairs, respectively (Fig. 1A, B). This is consistent with previous findings that methylation levels of meCpGs are primarily negatively associated with CTCF occupancy [13, 14].

Our analysis showed that meCpG-CTCF pairs with negative correlations tend to have lower CpG methylation levels and reduced CTCF occupancy compared to those with positive correlation (Fig. 1C, D). Additionally, meCpG sites negatively associated with CTCF binding are more likely to be located closer to the center of the corresponding CTCF binding sites (Fig. 1E and Additional file 5: Fig. S1E). In total, we obtained 6788 significantly associated meCpG-CTCF pairs for further examination of their modulation effects on eQTL (Additional file 1: Table S1).

### memo-eQTL mapping reveals hidden relationship between SNP and gene

**Fig. 2:**
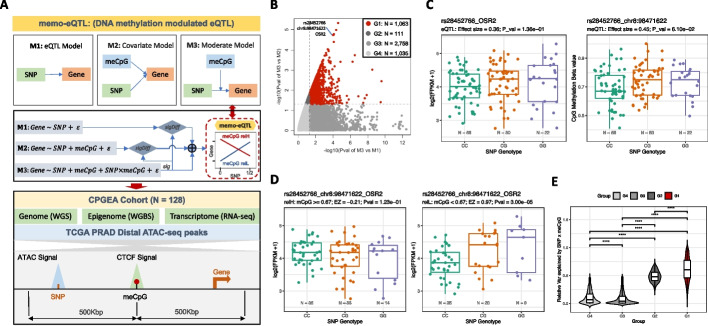
Mapping and characteristics of memo-eQTLs. **A** The framework of memo-eQTL mapping method and its implementation in the CPGEA cohort (sig: significant; sigDiff: significantly different; relH and relL refer to the subsamples with relatively high and low methylation levels at corresponding meCpG site, respectively). **B** Four different groups of SNP-meCpG-Gene combinations based on comparisons of M3 versus M1 and M3 versus M2 after requiring that M3 be significant. Note that combinations belonging to the group 1 (G1) are considered as memo-eQTLs. **C** Canonical eQTL (left) and meQTL (right) analysis for SNP rs28452766 with gene OSR2 and CpG site at chr8:98,471,622, respectively. **D** Visualization of selected memo-eQTL, depicting the relationship between rs28452766 and OSR2 in subsamples with relatively high (relH: Beta ≥ 0.67) and low (relL: Beta < 0.67) methylation levels at chr8:98,471,622. **E** The comparisons of the relative variance of gene expression can be explained by SNP × meCpG across groups G1-4 (Wilcoxon rank-sum two-sided test: ****p < 0.0001)

We introduced an extended eQTL method, named memo-eQTL, to systematically assess the modulation effects of these meCpGs, This method characterizes the modulation effect as the interaction between SNP and meCpG (SNP × meCpG) via a moderate model (M3). Subsequently, it requires comparisons against the covariate model (M2) and the standard eQTL model (M1) to determine the statistical significance of the modulation effect (Fig. 2A and “Methods”).

We conducted the memo-eQTL mapping in the Chinese Prostate Cancer Genome and Epigenome Atlas (CPGEA) cohort, which comprised matched whole-genome sequencing (WGS), RNA sequencing (RNA-seq), and WGBS data for 128 benign prostate samples [15] (Fig. 2A). Specifically, we pruned SNPs in high linkage disequilibrium (LD) and focused on 19,895 SNPs located in 14,374 ATAC-seq distal peak regions that were identified in prostate cancer [16] (Additional file 5: Fig. S2A and “Methods”). For meCpG sites, we pinpointed 1187 sites with variable methylation levels (Additional file 5: Fig. S2B and “Methods”), which were significantly correlated with CTCF binding as identified in Fig. 1A. Among the potential target genes, we preserved 14,520 protein-coding and lincRNA genes after filtering out lowly expressed ones (Median FPKM < 1, Additional file 5: Fig. S2C). Lastly, we conducted memo-eQTL analysis for 48,348 SNP-meCpG-Gene combinations, where the linear distance between the SNP and the Gene was up to 1 million base pairs. Importantly, we required the meCpG site to be located in between the paired SNP and Gene to simplify the possible modulating mechanisms (Fig. 2A and “Methods”).

In total, we identified 1063 memo-eQTLs, which not only displayed a statistically significant SNP × meCpG interaction (M3 versus M2), but also surpassed canonical eQTL models in performance (M3 versus M1) (Fig. 2B and Additional file 2: Table S2). Notably, only 93 of these memo-eQTLs were detected as eQTLs for the corresponding genes, and 81 as methylation quantitative trait loci (meQTLs) associated with matched meCpGs, all with *p*-values less than 0.05 (Additional file 2: Table S2). For instance, the SNP rs28452766 is neither an eQTL for OSR2 nor a meQTL for the CpG site at chr8:98,471,622. However, it is significantly associated with OSR2 expression levels in the subsamples with relatively low (relL) methylation levels at chr8:98,471,622 (Fig. 2B–D and “Methods”). This suggests the potential modulating capability of the meCpG site on the relationship between rs28452766 and OSR2 expression. Notably, none of the 1063 memo-eQTL SNP and gene pairs were reported as eQTL in the prostate samples from GTEx (dbGaP Accession phs000424.v8.p2). In addition, subsampling the GTEx prostate samples to the same sample size as the CPGEA cohort showed that the vast majority of the 40,740 SNP-gene pairs examined in memo-eQTL mapping did not exhibit significant associations (Additional file 5: Fig. S2D).

To further validate the credibility of our identified memo-eQTLs, we conducted permutation tests by randomly splitting 128 GTEx samples into DNA methylation relatively high (relH) and low (relL) groups 1000 times to simulate the modulation effects of meCpG (“Methods”). Remarkably, 350 out of the 375 memo-eQTLs significant in the relH group exhibit significantly lower *p*-values of eQTL model in comparison to random sample partitions (Additional file 5: Fig. S2E). A similar trend was observed for 329 out of the 344 memo-eQTLs that were significant in the relL group (Additional file 5: Fig. S2E). These results underscore the capability of memo-eQTL to uncover the intricate meCpG modulated interactions between SNPs and Genes that exceed random expectations. Moreover, recognizing that DNA methylation level variations could arise from different cell type compositions, leading to potential misidentification, we accounted for potential confounding effects by estimating the proportions of different cell types within our samples. Our analysis revealed that, on average, about 72% of the cells within our samples are non-immune (Additional file 2: Table S2), with low variation in cell type composition (Additional file 5: Fig. S2F, G and Additional file 2: Table S2). These results suggest that the variation in cell type composition within our sample was minimal, consistent with expectations for healthy samples. Furthermore, our analysis showed that the SNP × meCpG explained the most substantial portion of gene expression variance for memo-eQTLs compared to non-significant groups (Fig. 2B, E). In contrast, the SNP or meCpG alone tends to explain less gene expression variance for memo-eQTLs compared to other groups (Fig. 2B and Additional file 5: Fig. S2H). These findings further emphasize the unique modulation role of meCpG in gene expression captured by memo-eQTL mapping.

In conclusion, these results suggest that memo-eQTL mapping complements canonical eQTL and meQTL analyses, uncovering previously uncaptured relationships between genetic variant and gene expression modulated by DNA methylation.

### The characteristics of meCpGs, genes and SNPs involved in memo-eQTLs

**Fig. 3:**
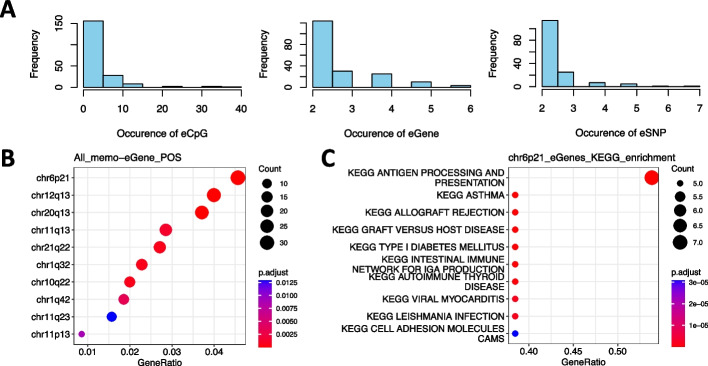
Characteristics eCpG, eGene, and eSNP for memo-eQTLs. **A** The occurrence of eCpG (left), eGene (middle), and eSNP (right) in 1063 memo-eQTLs. **B** Enrichment of eGenes in various chromosome regions. **C** Enriched KEGG pathways for eGenes located in chr6p21

Among the 1063 memo-eQTLs, there are 352, 749, and 847 unique meCpGs, genes, and SNPs, respectively. Hereafter, they are termed as eCpGs, eGenes, and eSNPs (Fig. 3A). Notably, an eCpG can modulate the relationships of up to 37 pairs of eSNP and eGene, and an eGene can also be associated with as many as six combinations of eCpG and eSNP, indicating possible dominance or additive modulation effects by eCpGs (Fig. 3A). To explore the biological processes and functions regulated by memo-eQTLs, we performed Gene Ontology (GO) enrichment analysis for all eGenes and found that they are enriched on chr6p21 and over-represented in several immune-related Kyoto Encyclopedia of Genes and Genomes (KEGG) pathways (Fig. 3B, C), especially in the antigen processing and presentation pathway, crucial for adaptive immunity [17]. Nevertheless, given the high linkage disequilibrium in the MHC region on chr6p21 [18], further investigations are needed to pinpoint which eGene corresponds to the associated eSNPs. It is worth noting that the eGene SRD5A3 was reported as a risk gene for prostate cancer in our transcriptome-wide association study (TWAS) analysis using two distinct prostate cancer GWAS studies (Additional file 5: Fig. S3A) [19–21]. These findings suggest that our memo-eQTLs can enhance the comprehension of genetic regulatory mechanisms underlying risk genes associated with specific traits and diseases.

Of the eSNPs identified, 63 were also reported as meQTLs, indicating that the same SNP could simultaneously influence both DNA methylation levels and gene expression (Additional file 2: Table S2 and Additional file 5: Fig. S3B). The intricate mechanism underlying this dual regulatory role on gene expression could involve modulation and mediation through changes of DNA methylation at corresponding CTCF binding sites. However, thorough understanding of these simultaneous effects necessitates further in-depth investigation. Moreover, among the 847 unique eSNPs, we found 30 have been previously reported to be associated with 19 types of traits or diseases, such as hypertension and Alzheimer’s disease in GWAS studies (Additional file 3: Table S3) [1]. An additional 206 eSNPs were found to be in high LD with significant risk SNPs in GWAS studies (“Methods”). These results underscore that memo-eQTLs can complement canonical eQTLs, aiding in interpreting associations between SNPs and traits or diseases detected by GWAS.

### eCpG-CTCF-based chromatin loop can either insulate or enhance the regulatory interaction between eSNP and eGene

**Fig. 4:**
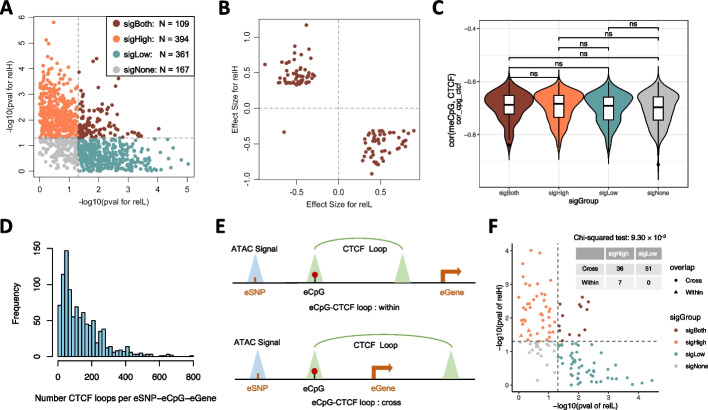
Mechanisms investigation for memo-eQTLs.** A** Stratified four subgroups of memo-eQTLs based on the *p*-values of M1 models for relatively high (relH) and low (relL) subsamples of the eCpG site, 0.05 was used as the significance cutoff. **B** The effect size and direction of eSNPs on eGenes in eCpG relH and relL subsamples for the sigBoth memo-eQTLs. **C** Comparisons of the Spearman correlation coefficients for meCpG-CTCF pairs across four memo-eQTL groups (Wilcoxon rank-sum two-sided test: ns: not significant). **D** The number of 22Rv1 HiChIP data derived CTCF loops that overlapped with the eSNP-eCpG-eGene loci. **E** The illustration plot of the overlapping patterns between eSNP-eCpG-eGene loci and eCpG-CTCF loops. **F** The overlapping patterns between the eSNP-eCpG-eGene loci and eCpG-CTCF loops derived from 22Rv1 HiChIP data for the four memo-eQTL groups (chi-squared test: *p* = 1.68 × 10.^−3^)

In a prior study, we demonstrated that a high CpG methylation level can impede CTCF binding and the formation of a 3D chromatin loop. This allows for cross-talk between a SNP and its target genes. Conversely, low CpG methylation levels correlate with increased CTCF binding and creation of the 3D loop, which acts as an insulator blocking the interplay between the SNP and target genes [7]. To systematically assess the underlying regulatory mechanism for memo-eQTLs, we categorized them into four groups based on whether significant associations between eSNPs and eGenes were observed in subsamples with either high or low eCpG methylation levels: sigHigh, sigLow, sigBoth, and sigNone memo-eQTLs (“Methods”). To simplify the categorization, we excluded the 32 memo-eQTLs with meCpG sites positively correlated with CTCF binding, resulting in 394 sigHigh, 361 sigLow, 109 sigBoth, and 167 sigNone memo-eQTLs (Fig. 4A).

Interestingly, we observed that eSNPs tend to have opposite relationships with eGenes in subsamples with relatively high and low eCpG methylation levels, particularly in the sigBoth memo-eQTLs (Fig. 4B and Additional file 5: Fig. S4A). This finding suggests that the meCpG modulation effect not only determines the presence of the interaction between genetic variant and gene expression but also alters the direction of their cross-talk. When delving into the distances among eSNP, eCpG, and eGene across four types of memo-eQTLs, we found no significant differences in the pairwise linear proximities (Additional file 5: Fig. S4B and Additional file 4: Table S4). Additionally, the correlations between meCpG and CTCF binding were consistent across all four groups (Fig. 4C). Together, these results suggest that eCpG primarily modulates the interplay between eSNP and eGene by influencing CTCF-based chromatin organization, thereby altering their spatial proximity.

To validate this hypothesis, we employed the CTCF-based 3D chromatin interaction data from two prostate cancer cell lines, 22Rv1 and VCaP, as well as the prostate epithelial cell line RWPE-1 to examine the spatial relationship among eCpG, eGene, and eSNP (“Methods”). In analyzing the 3D chromatin interaction data from 22RV1, we found that the eSNP-eCpG-eGene loci of 997 memo-eQTLs coincided with CTCF loops. Notably, 99.8% of these regions intersected with more than one loop (Fig. 4D). We further identified 756 eCpGs sites located in the anchor sites of 3879 CTCF loops, which allowed us to directly assess their modulation effects through 3D structure alteration. To simplify the analysis, we focused on 128 eCpGs that overlapped with a single CTCF loop anchor site, which we termed as eCpG-CTCF loop. There were only nine eCpG-CTCF loops fully embedded in corresponding eSNP-eCpG-eGene loci, while the remaining 119 eCpG-CTCF loops partially intersected with the eSNP-eCpG-eGene loci. These two groups were termed as “within” and “cross” eCpG-CTCF loops, respectively, as shown in Fig. 4E. We then extended the analysis using 3D chromatin interaction data from VCaP and RWPE-1. Overall, we identified 36, 16, and 5 sigHigh memo-eQTLs with Cross eCpG-CTCF loops derived from 22Rv1, VCaP, and RWPE-1, respectively (Fig. 4F, Additional file 5: Fig. S4C, D and Additional file 4: Table S4), suggesting the corresponding eCpGs may block the formation of CTCF loops, which could act as insulators for the interplay between eSNP and eGene. In contrast, we identified 3 and 23 sigLow memo-eQTLs with Within eCpG-CTCF loops derived from VCap and RWPE-1 (Additional file 5: Fig. S4C, D and Additional file 4: Table S4), implying the corresponding eCpGs might promote the formation of CTCF loops, which could enhance the interaction between the eSNP and eGene by bringing them physically closer. Although a distinct preference among the different types of memo-eQTLs for intersecting with eCpG-CTCF loops was not apparent (Fig. 4F and Additional file 5: Fig. S4D), we found that meCpG modulated CTCF-based chromatin 3D organization could either insulate or enhance the cross-talk between genetic variant and gene expression. Yet, these intricate mechanisms warrant further exploration.

## Discussion

Unlike canonical eQTL or meQTL, which examine the association between gene expression and genetic variant or DNA methylation alone, our previous research revealed that DNA methylation can modulate the interplay between genetic variants and gene expression. This is achieved by dichotomizing the population into high and low methylation groups based on specific CpG site methylation levels [7]. To evaluate this type of modulation effect more broadly, we developed a sophisticated method named memo-eQTL. This approach incorporates the genetic variant and DNA methylation, along with their interaction, into a multiple regression model. The statistical significance of the DNA methylation modulation effect is determined by comparing this model with and without the interaction [10, 11]. We used the original continuous methylation levels instead of dichotomizing it as a categorical variable in our previous study. This enabled us to investigate the effects of genotypes at varying methylation levels. To accentuate the differing effects of the SNP on gene expression at relatively high and low CpG methylation levels, we pinpointed the optimal separation reflecting the more pronounced modulation effects. Moreover, our memo-eQTL analysis unveiled not only eSNPs linked to GWAS traits or diseases, but also highlighted memo-eQTLs and eQTLs with shared eGenes. Such shared eGenes have the potential to serve as risk genes for specific traits and diseases, as demonstrated through the TWAS analysis. Collectively, our memo-eQTLs contribute significantly to enhancing our understanding of the intricate genetic regulatory mechanisms underlying risk-associated genetic variants and their impact on gene expression in the context of specific traits and diseases.

We first examined the correlation between CTCF occupancy and CpG methylation across 26 cell types and tissues. Although about 97.8% of significant pairs are negatively correlated, a small subset of pairs exhibit positive relationships, which has also been observed in previous studies [13]. These results confirmed the overall inverse relationship between the CpG methylation and CTCF binding. However, the presence of this subset of positively correlated pairs suggests that additional factors may be involved, such as cofactors that interact with CTCF and selectively affect the methylation status at these binding sites [22, 23]. There are 32 memo-eQTLs that are also engaged with positively correlated meCpG-CTCF pairs, which provide an alternative meCpG modulation model for the interplay between the eSNP and eGene.

Comparing our memo-eQTLs with GTEx eQTLs, we consistently observed that the vast majority of SNP-Gene pairs were not significantly associated with canonical eQTL. However, a noteworthy discrepancy arose when we examined the significant SNP-Gene pairs (~ 2000), revealing a mere 5% overlap detected in both cohorts. This variance is likely primarily attributed to the inherent demographic differences between the two cohorts. The CPGEA cohort we utilized comprises 128 matched normal samples obtained from Chinese prostate cancer patients, all aged over 50 [15]. In contrast, the GTEx cohort predominantly consists of the normal prostate tissue samples from healthy participants of European ancestry (> 80%), with ~ 35% being younger than 50 (dbGaP Accession phs000424.v8.p2). These data underscore the significance of considering demographic factors when conducting memo-eQTL and eQTL analyses and/or comparisons, as such factors can substantially influence the results.

Considering the potential impact of DNA methylation variations from different cell types within the tissue samples, it is important to conduct cell type estimation to assess this influence. In this study, we turned to gene expression-based tools such as EPIC [24] and xCell [25], which concluded that the variation in cell type composition within our sample is relatively stable, aligning well with our expectation for healthy samples. While the DNA methylation-based methods like EpiDISH [26] and MethylCIBERSORT [27] are also powerful tools for cell type estimation, their effectiveness in our study was limited due to the lack of overlapping CpG sites with reference DNA methylation signatures. Moreover, alternative methods like ReFACTor [28] and RUVm [29] have also been proposed to correct for such cell type epigenome heterogeneity. However, it is essential to recognize that these methods vary in their performance, and no single approach is universally optimal, especially for complex tissue samples like tumors [30]. Looking ahead, the emerging single-cell omics data presents an opportunity for robust cell type imputation or characterization. Therefore, it is of great interest to adapt our approach in a cell type-specific manner in subsequent research.

Since most of the eCpGs are negatively associated with CTCF binding, we assumed that the sigHigh memo-eQTLs would preferentially partially intersect with CTCF looping, as a result, inhibiting the CTCF-based loop formation can blockade the interplay between eSNP and eGene. Conversely, sigLow memo-eQTLs would be more likely fully located in the CTCF loop, allowing for increased eSNP and eGene interactions since the loop would bring them physically closer. However, we did not observe clear preference between sigHigh and sigLow memo-eQTLs regarding the overlapping pattern with eCpG-CTCF loops. One possible explanation is that the vast majority of memo-eQTL eSNP-eCpG-eGene regions overlapped with multiple CTCF loops, complicating the task of distinguishing 3D structure differences among various memo-eQTLs groups. In addition, it is worth noting that we had successfully validated the meCpG modulated insulation mechanism at MYC locus in our previous study [7], which paves the way to delve deeper into these intricate mechanisms in subsequent studies.

## Conclusions

The memo-eQTL method offers a valuable tool for identifying DNA methylation modulated eQTLs that are often missed by canonical eQTL analysis, thereby allowing for the discovery of novel target genes that are associated with genetic variants and diseases. We found that DNA methylation modulated CTCF-based chromatin 3D organization can either insulate or enhance the cross-talk between genetic variant and gene expression. We anticipate that as more 3D chromatin data becomes available, our understanding of these regulatory mechanisms will continue to improve. Overall, our findings suggest that the memo-eQTL method, coupled with the study of chromatin 3D organization, presents a complementary framework for identifying and understanding the complex regulatory processes underlying genetic variation and gene expression.

## Methods

### Correlation between CpG methylation and CTCF binding

To examine the relationship between CpG methylation and CTCF binding, we gathered a total of 95,887 CTCF binding sites from 26 human cell lines or tissues (Additional file 5: Fig. S1A), along with the methylation levels of 1,188,556 CpG dinucleotides located on these CTCF binding sites, from the ENCODE portal [12]. For meCpG sites, we required at least 10 cell lines or tissues with more than 20 WGBS reads, as well as an Interquartile Range (IQR) of Beta values greater than 0.1. For the CTCF binding intensity, we required an IQR greater than 1 (Additional file 5: Fig. S1B). Then, the Spearman correlation coefficient (SCC) and *p*-value for each CpG site’s methylation levels and corresponding CTCF binding intensity were calculated, and the *p*-values were adjusted for multiplicity using the BenJamini–Hochberg procedure. Lastly, a significant association between meCpG and CTCF binding was defined when the absolute SCC was greater than 0.5 and the Padj was smaller than 0.05 (Additional file 1: Table S1).

### SNP-CpG-Gene combinations for memo-eQTL mapping

Using the processed WGS, WGBS, and RNA-seq data for 128 benign prostate samples from the CPGEA cohort [15], we extracted genotype information for SNPs with rsIDs in dbSNP (build 151) and a minor allele frequency (MAF) greater than 0.05, and removed SNPs in high LD (squared correlation ≥ 0.8) using the PLINK pruning function (www.cog-genomics.org/plink/1.9/). Given the potentially functional capability, we focused on 19,895 pruned SNPs located in 14,374 ATAC-seq distal peak regions that were identified in prostate cancer [16] (Additional file 5: Fig. S2A). For DNA methylation data, we focused on 1187 meCpG sites with average methylation levels (Beta value) within the range of [0.25, 0.75] and IQRs greater than 0.1 (Additional file 5: Fig. S2B), and that were also found significantly correlated with CTCF binding intensity in Fig. 1A. As for potential target genes, we retained 14,520 protein-coding and lincRNA genes after filtering out lowly expressed ones (Median FPKM < 1) and inverse normal transformed their expression levels (Additional file 5: Fig. S2C). We then searched for all possible SNP-CpG-Gene combinations within a 1 million base pair window size. Importantly, we required the CpG site to be located in between of paired SNP and gene to ensure possible modulating effects (Fig. 2A).

### memo-eQTL mapping, assessment, and grouping

Three models were built for memo-eQTL mapping: M1, the standard eQTL model, determines the effect of SNP on gene expression; M2, the covariate model, examines the additive marginal effects of SNP and DNA methylation on gene expression; and M3, the moderate model, characterizes the DNA methylation modulation effect as the interaction between the SNP and meCpG (SNP × meCpG) on top of M2. These models were built for all 48,348 SNP-meCpG-Gene combinations.

$$\begin{array}{c}\mathbf{M1}:\mathrm{ Gene }= {\mathrm{\alpha }}_{1} +{\upbeta }_{1}\mathrm{ \,SNP }+ {\upvarepsilon }_{1}\\ \mathbf{M2}:\mathrm{ Gene }= {\mathrm{\alpha }}_{2}+ {\upbeta }_{21}\mathrm{ \,SNP }+ {\upbeta }_{22}\mathrm{\, meCpG}+ {\upvarepsilon }_{2}\\ \mathbf{M3}:\mathrm{ Gene }= {\mathrm{\alpha }}_{3} + {\upbeta }_{31}\mathrm{ \,SNP }+ {\upbeta }_{32}\mathrm{ \,meCpG }+{\upbeta }_{33} \times \mathrm{ \,meCpG }+ {\upvarepsilon }_{3}\end{array}$$

For memo-eQTLs, first, we required that M3 is significant, and the contribution of the SNP × meCpG interaction is also significant by comparing the M3 and M2 models [10, 11]. To ensure that the significant SNP × meCpG interaction was driving the presence or enhancement of the relationship between SNP and gene expression, we also required that the comparison between M3 and M1 models be significant (Fig. A, B). Specifically, the likelihood ratio test (LRT) [31] was employed to compare the two models, and a *p*-value threshold of 0.05 was used to determine statistical significance.

To assess the performance of using our *p*-value-based method and canonical FDR-based method in identifying significant memo-eQTLs, we devised an artificial dataset for thorough analysis. This dataset employed the identical SNP and meCpG data for all tested SNP-CpG-Gene combinations from our presented study, while the gene express data (*y*) was deliberately generated through randomization using the following formula:

$$y=\beta_0+\beta_1SNP+\beta_2\,meCpG+\beta_3\,SNP\times meCpG+\epsilon,$$

where different *β* values combination were randomly selected from the Additional file 2: Table S2, and $\epsilon \sim N(0, {\sigma }^{2})$ accounts of the normal distributed error term with the same sample size (*N* = 128). Subsequently, we conducted the memo-eQTL mapping for all SNP-CpG-Gene combinations using this artificial dataset. The real memo-eQTL was defined as when ${\beta }_{3}\ne 0$, and the predicted memo-eQTL was determined using both *p*-value-based and FDR-based methods. Lastly, both F1 scores (as defined below) and the count of the True Positives were examined to evaluate the performance of both methods.

$$F1 = \frac{2 \,* \,True \,Positive}{2\,* \,True \,Positive \,+ \,False \,Positive \,+ \,False \,Negative}$$

If we were to apply the FDR correction solely to the M3 versus M2 to ascertain memo-eQTLs, we achieved a comparable F1 score (F1 = 0.31) to that achieved by our proposed *p*-value-based method (F1 = 0.34). However, it is noteworthy that the *p*-value-based method yields a higher number of true positive memo-eQTLs (24,179 vs 12,260) when compared to the FDR-based method.

To visualize the effects of SNP × meCpG for memo-eQTL, we dichotomized the 128 samples based on the optimal threshold of meCpG levels that distinguished the M1 models in meCpG relatively high (relH) and low (relL) subsamples the most. This threshold was searched within the range between the lower (Q1) and higher (Q3) quartiles of meCpG levels (Fig. 2D). Furthermore, we computed the relative explained variances of gene expression by SNP, meCpG, and SNP × meCpG in M3 across the G1-4 groups by normalizing the individually explained variance to their sum (Fig. 2E and Additional file 5: Fig. S2H). Lastly, the memo-eQTLs were split into four groups (sigHigh, sigLow, sigBoth, and sigNone) based on the statistical significance of the M1 models in relH and relL subsamples (Fig. 4A). Specifically, we examined whether these models were significant (*p*-value < 0.05) in either or both relH and relL subsamples.

### Comparison with GTEx eQTLs

Given the specific prerequisites outlined in Fig. 2A for our memo-eQTL mapping entails for SNP, meCpG, and Gene combinations, we focused on 40,740 SNP-Gene pairs examined within our memo-eQTL framework to ensure a fair comparison with GTEx eQTLs. Specifically, we initially performed random sampling of 128 GETX prostate samples with matched gene expression and genotype data on 10 separate occasions (dbGaP Accession phs000424.v8.p2). Within these samplings, we identified significant associated SNP-Gene pairs using M1 with a *p*-value threshold of less than 0.05. Furthermore, to enhance credibility of the memo-eQTL, we conducted the permutation tests. These tests involved randomly dividing 128 GTEx samples into two groups 1000 times for each sigHigh and sigLow memo-eQTLs, assuming one group with relatively high (relH) DNA methylation levels and the other with relatively low levels (relL) to mimic the modulation effects of meCpG. Notably, the partition of the relH and relL groups is consistent with the group sizes of the corresponding memo-eQTLs in each simulation.

### Cell type estimation and TWAS analysis

To evaluate the inherent variability of DNA methylation levels and variations across different cell types, both EPIC [24] and xCell [25] were utilized to estimate the cell type proportions using the gene expression data. And the coefficient of variation (CV) was calculated to assess the variability of cell type compositions across samples. To identify potential risk memo-eQTL eGenes underlying specific complex traits and disease, we conducted the transcriptome-wide association study (TWAS) [32] using the tools suite FUSION (http://gusevlab.org/projects/fusion/), where we leveraged pre-computed gene expression weights derived from GTEx prostate data and GWAS summary statistics from two distinct prostate cancer GWAS studies [19, 21].

### GWAS SNPs and their high LD SNPs

All significant SNP-trait associations were downloaded from the GWAS catalog [1], and only those SNPs with rsIDs were examined in our analysis. To identify SNPs in high LD, we used genotype data for the East Asian (EAS) population from the 1000 Genomes phase3 data [33]. To be specific, the PLINK v1.9 (www.cog-genomics.org/plink/1.9/) was employed to scan all SNP pairs within the query SNP’s centralized 1 million base pair window, and SNPs with an R2 value greater than 0.8 were considered in high LD.

### eSNP, eGene, and eCpG linear and spatial distance examination

To determine the linear distance between the eSNP, eCpG, and eGene, we calculated the pairwise distances between any two of them. The distance to eGene was measured based on the transcription start site (TSS). All distances were measured strandless since meCpG is strandless, and negative distance indicates the location of the former element on the left side. To explore the chromatin 3D structures, we called CTCF-associated chromatin loops from CTCF HiChIP data in VCaP and 22Rv1 cells (GSE172498) using HiCUP (v0.7.2) and hichipper (v0.7.7) pipelines. Additionally, we utilized CTCF ChIA-PET data from the normal prostate cell line (RWPE-1) [34] and focused on the 5068 strongest CTCF-based chromatin loops with at least 15 PET reads. To simplify the investigation of mechanism, we focused on those eCpGs located in the anchor site of a single CTCF loop as derived above.

### Statistical analysis

The comparison of continuous variables between groups was conducted using the Wilcoxon rank-sum two-sided test or the Kolmogorov–Smirnov test, while the comparison of categorical variables was conducted using chi-squared test. All statistical significance was provided, and the results were considered significant if the *p*-value was less than 0.05.

### Supplementary Information

**Additional file 1: Table S1.** Significantly correlated meCpG-CTCF pairs. All significantly correlated meCpG-CTCF pairs (sheet 1) and the most significantly correlated meCpG-CTCF pair per each CTCF binding site (sheet 2).

**Additional file 2: Table S2.** memo-eQTL mapping results and assessment. Different β values combinations used for memo-eQTL mapping simulation (sheet 1). Meta information for 1,063 memo-eQTLs (sheet 2). The estimated cell type proportions by EPIC (sheet 3) and xCell (sheet 4) using gene expression data.

**Additional file 3: Table S3.** memo-eQTL eSNPs that overlap with GWAS risk SNPs.

**Additional file 4: Table S4.** Linear and spatial patterns for memo-eQTLs. Pairwise linear distances between eSNP, eCpG and eGene for 1,031 memo-eQTLs (sheet 1). The spatial overlapping patterns between the eSNP-eCpG-eGene loci and eCpG-CTCF loops derived from 22Rv1(sheet 2), VCaP (sheet 3) HiChIP data and RWPE-1 ChIA-PET data (sheet 4).

**Additional file 5:** **Figure S1-S4.**

**Additional file 6:** Peer review history.

## Data Availability

The analyses were primarily performed using R 4.0.3 (http://CRAN.R-project.org, R Foundation, Vienna, Austria). All source data and code have been made publicly available on GitHub: https://github.com/HansenHeLab/memo-eQTL_Data_Codes [35] and Zenodo: https://zenodo.org/records/10151167  [36]. Both repositories are released under the MIT license.
