# Supplementary material for: DNA methylation modulated genetic variant effect on gene transcriptional regulation
Source: Genome Biol. 2023 Dec 8;24:285. doi: 10.1186/s13059-023-03130-5 (PMC10709945; doi:10.1186/s13059-023-03130-5)
Supplement: Supplementary file 5 — Additional file 5: Figure S1-S4. [file 13059_2023_3130_MOESM5_ESM.docx]

**Figure S1: The characteristics of meCpG and CTCF binding sites. A)** The number of CTCF peaks for 26 different tissue and cell line samples from the ENCODE. **B)** The distributions of the interquartile range (IQR) for meCpG methylation levels (left) and CTCF-binding intensity (right). **C)** Correlation coefficients and statistical significance for all meCpG-CTCF pairs. Neg, Pos and notSig refer to negatively correlated, positively correlated and not significantly correlated meCpG-CTCF pairs, respectively. **D)** Distribution of the number of meCpG sites per CTCF-binding site for significantly correlated meCpG-CTCF pairs. **E)** Comparisons of distance between meCpG site and the center of CTCF-binding site among three different groups (Kolmogorov-Smirnov test: Pos vs Neg: p = 6.73 × 10^-6^; Pos vs notSig: p = 1.84 × 10^-2^; Neg vs notSig: p < 2.20 × 10^-16^).

**Figure S2: The preparation and characteristics of memo-eQTL . A)** The distribution of the number SNPs located in ATAC peaks after filtering and pruning. **B)** The distribution of the median methylation levels for measured CpG sites across 128 samples. **C)** The distribution of the median gene expression levels for protein-coding and lincRNA genes across 128 samples. **D)** Overlapping of non-significant associated SNP-Gene pairs between CPGEA (Ref) and 10 random sampling occasions from GTEx (Sample_1-10). **E)** The distribution of the p values for permutation tests emulating the meCpG modulation effects for memo-eQTLs significant only in the relH (right) and relL (left) subsamples. The relationship and the CV and Median of the all cell type fractions derived from EPIC (**F**) and xCell (**G**) estimation. **H)** The comparisons of relative variance of gene expression can be explained by SNP (left) and meCpG (right) alone across groups G1-4 (Wilcoxon rank-sum two-sided test: ****p < 0.0001).

**Figure S3: The memo-eQTLs help explain GWAS risk SNP. A)** Visualization of memo-eQTLs, eQTLs and meQTLs for prostate cancer risk Gene SRD5A3. **B)** An example of a memo-eQTL that was also identified as a meQTL.

**Figure S4: The memo-eQTLs help explain GWAS risk SNP. A)** The effect size and direction of eSNPs on eGenes in eCpG relH and relL subsamples for the sigHigh, sigLow and sigNone memo-eQTLs. **B)** The distribution and comparison of distances between eSNP and eGene (left), eSNP and eCpG (middle) and eCpG and eGene (right) for the four memo-eQTL groups. Negative distances indicate the former element is on the left side of the latter element (Kolmogorov-Smirnov test: left: p = 1.04 × 10^-1^; middle: p = 5.81 × 10^-1^; left: p = 2.59 × 10^-1^). **C)** The number of VCaP (top) and RWPE-1 (bottom) cell lines data derived CTCF loops that overlapped with the eSNP-eCpG-eGene loci. **E)** The overlapping patterns between the eSNP-eCpG-eGene loci and eCpG-CTCF loops derived from VCaP (left) and RWPE-1 (right) 3D chromatin interaction data for the four memo-eQTL groups (Chi-squared test: VCaP, p = 0.25; RWPE-1, p = 1).
