# Supplementary material for: DNA methylation modulated genetic variant effect on gene transcriptional regulation
Source: Genome Biol. 2023 Dec 8;24:285. doi: 10.1186/s13059-023-03130-5 (PMC10709945; doi:10.1186/s13059-023-03130-5)
Supplement: Supplementary file 6 — Additional file 6: Peer review history. [file 13059_2023_3130_MOESM6_ESM.docx]

Review History

**First round of review**

**Reviewer 1**

**Were you able to assess all statistics in the manuscript, including the appropriateness of statistical tests used?**

Yes, and I have assessed the statistics in my report.

**Comments to author:**

The paper presents a novel approach to the study of expression quantitative trait locus (eQTL) analysis by introducing DNA methylation modulation(memo-eQTL) based on their early single gene (Myc) example.  memo-eQTL analysis on  128 normal prostate samples  resulted in the discovery of 1,731 memo-eQTLs, most of which have not been reported previously as eQTLs. This discovery is of great significance as it paves the way for better understanding of how genetic variations can lead to traits or diseases. The paper is well-structured, and the methodology is clearly explained.

However, the study would have benefited from a more detailed comparison of the conventional eQTL as well as the implications of these findings for the broader field of genetics.

1. They need to perform a fair comparison between memo-eQTL and GTEx eQTL with similar sample size. This can be done through downsampling of GTEx cohort. Please also discuss the difference between Chinese cohort and GTEx cohort.

2. The number of memo-eQTLs (1731) seems to be very low comparing with GTEx eQTLs. It's unclear how many additional disease/trait associated risk genes can be explained by memo-eQTLs alone. In another word, although memo-eQTLs were different from eQTLs, they might example the same risk genes.  The authors need to usecollocation and/or TWAS to answer this question.

**Reviewer 2**

**Were you able to assess all statistics in the manuscript, including the appropriateness of statistical tests used?**

Yes, and I have assessed the statistics in my report.

**Comments to author:**

In this manuscript, Zeng et al. developed an extended eQTL mapping method named memo-eQTL and applied it to systematically identify the memo-eQTL in 128 normal prostate samples. They discovered 1,731 memo-eQTL and found that the interaction between SNP and gene expression by altering CTCF-based chromatin 3D structure. However, some of the methods and the results require further discussion. 

Firstly, the authors emphasized that their analysis would complement previous eQTL methods and found some previously undiscovered eQTLs or meQTLs. But, the main reason for this may be not caused by their good testing ability, it is likely that they have a small number of initial SNP input (they only analyzed 19,895 SNPs located in 14,374 ATAC-seq distal peak regions), which will make it easier for them to obtain more significant sites when performing FDR correction. In GTEx, because of FDR analysis, the raw P values of eQTLs should be less than 10-5, but in this study, many P values are not very significant. For example in Figure 3D and 3E, Figure 3S, some P values are only around 0.1.

Secondly, in the first part of the results, the authors identified the meCpG associated with CTCF occupancy. As we know, epigenetic markers have strong tissue specificity. The users used only 26 human cell lines or tissues and performed the correlation analyses using the Pearson Correlation Coefficient, which may result in lots of false positive results. For example, in Figure 1b, the distribution of CTCF is not Normal distribution, suggesting that the Spearman correlation should be used instead of Pearson correlation. Additionally, the chosen cutoff (the Interquartile Range (IQR) of Beta values greater than 0) is a very loose threshold.

Thirdly, I suggest that the authors design some experiments to validate the modulation effect of meCpG, for example, the rSNP of rs77216612.

Overall, the model presented in this article is too simplistic and lacks experimental support. It does not meet the GB standard.

**Reviewer 3**

**Were you able to assess all statistics in the manuscript, including the appropriateness of statistical tests used?**

Yes, and I have assessed the statistics in my report.

**Comments to author:**

Zeng et al. introduced the concept of memo-eQTLs, a novel type of expression quantitative trait loci (eQTLs) regulated by DNA methylation of CTCF-binding sites. The authors revealed the existence of a previously overlooked group of eQTLs that would not have been detected without considering the statistical interaction between methylation and single nucleotide polymorphisms (SNPs). This submission emphasizes the intricate nature of eQTL identification and provides profound insights into the interplay between the genome and epigenome. Please find some minor comments below:

Applicability of the framework to other omics: DNA methylation variations can reflect differences in cell types or 3D genome structures. Can this analytical framework be adapted to investigate cell type-specific eQTLs or eQTLs specific to 3D looping?

I guess much of the challenge of memo-QTL is from the limitation of data to tissues with variable cell type composition, leading to methylation change. Assuming proper control of cell types, how many of the memo-QTLs would have been identified as eQTLs?

Independence assumption of methylation and SNP: The search for memo-QTLs began by focusing only on cases where all combinations of alleles and methylation states were present, automatically excluding cases where the SNP acted as a full meQTL. Is it possible for a SNP to simultaneously function as both an meQTL and a memo-QTL?

**Authors Response**

**Point-by-point responses to the reviewers’ comments:**

# We thank all the three reviewers for their careful reading of our manuscript, and their thoughtful feedback. We have addressed all the comments with additional analyses. Please find below our responses to each comment in blue.

# Reviewer #1.

The paper presents a novel approach to the study of expression quantitative trait locus (eQTL) analysis by introducing DNA methylation modulation(memo-eQTL) based on their early single gene (Myc) example.  memo-eQTL analysis on  128 normal prostate samples  resulted in the discovery of 1,731 memo-eQTLs, most of which have not been reported previously as eQTLs. This discovery is of great significance as it paves the way for better understanding of how genetic variations can lead to traits or diseases. The paper is well-structured, and the methodology is clearly explained.

However, the study would have benefited from a more detailed comparison of the conventional eQTL as well as the implications of these findings for the broader field of genetics.

We are grateful to the reviewer for recognizing the novelty of our work and providing insightful suggestions, which have greatly enhanced and strengthened our manuscript.

**Q1:** They need to perform a fair comparison between memo-eQTL and GTEx eQTL with similar sample size. This can be done through downsampling of the GTEx cohort. Please also discuss the difference between the Chinese cohort and the GTEx cohort.

We thank the reviewer for providing this insightful suggestion. Given the specific prerequisites outlined in **Figure 2A** for our memo-eQTL mapping, which entail combinations of SNP, meCpG and Genes, we focused on 40,740 SNP-Gene pairs examined within our memo-eQTL framework, to ensure a fair comparison with GTEx eQTLs.

To conduct this comparison, we randomly sampled 128 GETX prostate samples on 10 separate occasions. Although we identified approximately 2,000 significant associated SNP-Gene pairs across all occasions (p < 0.05), only around 5% of these pairs overlapped with those detected within the CPGEA cohort (**Reviewer** **Figure 1A**). This discrepancy could largely be attributed to the inherent differences between the cohorts. Specifically, the CPGEA cohort consists of 128 matched normal samples from localized prostate cancer patients in China, all aged over 50 [[1]](https://paperpile.com/c/v7YzmZ/7XCA). In contrast, the GTEx cohort is mainly composed of normal prostate tissue samples from healthy European ancestry participants (> 80%), with about 35% being younger than 50 (dbGaP Accession phs000424.v8.p2). Nevertheless, we consistently observed that the vast majority of SNP-Gene pairs did not display significant associations in canonical eQTL analysis in both cohorts (**Reviewer** **Figure 1B,** which has now been included as new **Fig. S2D**).

Furthermore, to enhance the credibility of the memo-eQTL, we conducted permutation tests. Specifically, we randomly divided the 128 GTEx samples into DNA methylation relatively high (relH) and low (relL) groups 1,000 times to simulate the modulation effects of meCpG. Notably, 350 out of 375 sigHigh memo-eQTLs exhibit significantly lower p-values in the eQTL model for the relH group compared to random sample partitions (**Reviewer Figure 1C,** also presented in new **Fig. S2E**). A similar trend was observed for the 329 out of 344 sigLow memo-eQTLs (**Reviewer Figure 1D,** also included in new **Fig. S2E**).

These results suggest that memo-eQTL has the ability to uncover the intricate meCpG modulated interactions between SNPs and Genes, which are not merely coincidental. We have incorporated these comparative results, along with the differences between the CPGEA and CTEx cohorts, into the **Results** and **Discussion** sections of the revised manuscript. The detailed comparisons have also been added to the **Methods** section.

**Reviewer Figure 1. Comparisons of eQTL between the CPGEA and GTEx cohorts**. Overlap of significant (**A**) and non-significant (**B**) associated SNP-Gene pairs between CPGEA (Ref) and 10 random sampling occasions from GTEx (Sample_1-10). Distribution of p-values from permutation tests simulating the meCpG modulation effects in sigHigh (**C**) and sigLow memo-eQTLs (**D**).

**Q2:** The number of memo-eQTLs (1731) seems to be very low comparing with GTEx eQTLs. It's unclear how many additional disease/trait associated risk genes can be explained by memo-eQTLs alone. In another word, although memo-eQTLs were different from eQTLs, they might example the same risk genes.  The authors need to use collocation and/or TWAS to answer this question.

We would like to express our gratitude to the reviewer for this great suggestion. The relatively lower number of memo-eQTLs we identified, compared to GTEx eQTLs, can be attributed, at least in part, to the more stringent prerequisites detailed in **Figure 2A** for our memo-eQTL mapping.

Moreover, our analysis led us to the identification of 30 eSNPs, which have previously been linked to an array of 19 traits or diseases in various GWAS studies. The unique feature of our memo-eQTLs, requiring meCpG modulation, renders the direct identification of causal genetic variants through collocation analysis unfeasible [[2]](https://paperpile.com/c/v7YzmZ/zDaa). However, we did pinpoint 188 eGenes shared between our memo-eQTLs and the GTEx eQTLs in the prostate. As the reviewer suggested, we performed a TWAS analysis [[3]](https://paperpile.com/c/v7YzmZ/u2aH) using two distinct prostate cancer GWAS studies [[4,5]](https://paperpile.com/c/v7YzmZ/EeWp+rNyL), and identified the shared eGene SRD5A3 as a risk gene for prostate cancer. These findings suggest that our memo-eQTLs can enhance our understanding of the genetic regulatory mechanisms underlying risk genes associated with specific traits and diseases.

We are deeply grateful to the reviewer for this insightful perspective. The corresponding results and a discussion has also been incorporated into the revised manuscript.

# Reviewer #2.

In this manuscript, Zeng et al. developed an extended eQTL mapping method named memo-eQTL and applied it to systematically identify the memo-eQTL in 128 normal prostate samples. They discovered 1,731 memo-eQTL and found that the interaction between SNP and gene expression by altering CTCF-based chromatin 3D structure. However, some of the methods and the results require further discussion.

Overall, the model presented in this article is too simplistic and lacks experimental support. It does not meet the GB standard.

We appreciate the valuable feedback from the reviewer. We have made significant revisions to strengthen the methods and conclusion of our manuscript. We hope the reviewer finds the revised manuscript suitable for publication in Genome Biology.

**Q1:** Firstly, the authors emphasized that their analysis would complement previous eQTL methods and found some previously undiscovered eQTLs or meQTLs. But, the main reason for this may be not caused by their good testing ability, it is likely that they have a small number of initial SNP input (they only analyzed 19,895 SNPs located in 14,374 ATAC-seq distal peak regions), which will make it easier for them to obtain more significant sites when performing FDR correction. In GTEx, because of FDR analysis, the raw P values of eQTLs should be less than 10-5, but in this study, many P values are not very significant. For example in Figure 3D and 3E, Figure 3S, some P values are only around 0.1.

We thank the reviewer for the comments. The primary objective of this study is to introduce the concept of considering the effects of interactions between SNPs and meCpG sites on gene expression regulation. These effects aren’t captured by canonical eQTL and meQTL analyses.

Regarding the selection of SNPs located in ATAC-seq peaks, our intention was to ensure their potential for genetic regulation. For the memo-eQTL mapping, multiple prerequisites and comparisons are required as outlined in **Figure 2A and 2B**. Specifically, we identified memo-eQTLs by ensuring that the **M3, M3** vs **M2**, and **M3** vs **M1** comparisons were all statistically significant (p-value < 0.05). This approach was chosen based on simulation studies. If we apply the FDR correction only to **M3** vs **M2** for memo-eQTLs determination, we would achieve a comparable F1 score (F1 = 0.31) as our proposed p-values-based method (F1 = 0.34). However, it’s noteworthy that our p-values-based method yields a higher number of true positive memo-eQTLs (24,179 vs 12,260) when compared to the FDR-based method. Detailed information regarding this simulation can be found in the **Reviewer Supplementary** section (also included in the **Methods** section) appended at the end of response letter.

Regarding the original **Figure 3D, 3E** and **3S**, the bottom panes depict the canonical eQTL (left) and meQTL (right), which aren’t necessarily significant (as shown in new **Fig 2C**, and **Fig. S3**). In fact, we observed that most of our memo-eQTLs are not detected as either canonical eQTL and meQTLs even without FDR correction in the same cohort. In contrast, the top panes illustrate the memo-eQTL by dividing the samples into relatively High and low meCpG groups. As demonstrated in **Figure 4A**, a memo-eQTL can be significant in either, both, or neither group.

We appreciate the review for this insightful perspective, and we have added the detailed simulation process to the **Methods** section.

**Q2:** Secondly, in the first part of the results, the authors identified the meCpG associated with CTCF occupancy. As we know, epigenetic markers have strong tissue specificity. The users used only 26 human cell lines or tissues and performed the correlation analyses using the Pearson Correlation Coefficient, which may result in lots of false positive results. For example, in Figure 1b, the distribution of CTCF is not Normal distribution, suggesting that the Spearman correlation should be used instead of Pearson correlation. Additionally, the chosen cutoff (the Interquartile Range (IQR) of Beta values greater than 0) is a very loose threshold.

We appreciate the reviewer for providing these constructive suggestions. We have revised the criteria, and applied a stringent requirement for the IQR of meCpG  to be greater than 0.1, resulting in 444,364 out of the initial 741, 933 meCpG-CTCF pairs remaining for the correlation testing. As suggested by the reviewer, we have used Spearman correlation testing for our revised manuscript (with updates in **Figure 1** and **Figure S1**). Notably, around 75% CTCF binding sites that display significant correlation with meCpG methylation levels via Spearman correlation testing also maintain consistent correlations when assessed using Pearson correlation, based on the same criteria (**Reviewer Figure 2A, B**). Additionally, for the overlapped correlated meCpG-CTCF pairs, the Pearson and Spearman correlation coefficients exhibit strong concordance (**Reviewer Figure 2C**). To reinforce the reliability of our subsequent findings, all related analyses in the manuscript have been revised accordingly to reflect these changes.

**Reviewer Figure 2**. The correlation between meCpG and CTCF. **A**) The most significantly correlated meCpG and CTCF per CTCF site using the Person correlation testing, with a stringent meCpG cutoff (IQR > 0.1). **B**) The fraction of overlapped CTCF sites, meCpG and meCpG-CTCF exhibiting significant correlations in Spearman testing, compared to the results of Pearson correlation testing. **C**) The correlation coefficients for the significantly correlated meCpG-CTCF pairs across both correlation tests.

**Q3:** Thirdly, I suggest that the authors design some experiments to validate the modulation effect of meCpG, for example, the rSNP of rs77216612.

We thank the reviewer for this valuable suggestion. Thanks to the constructive and insightful suggestions from all reviewers, we have refined our method and provided additional evidence to enhance the credibility of the modulation effect of meCpG.  Specifically, **1)** we have utilized the Spearman Correlation test as the reviewer suggested to ensure that meCpG robustly correlated with CTCF binding. This strengthens the foundation of our modulation effect analysis; **2)** we have confirmed that the observed modulation effects are not occurring by chance by conducting permutation tests (as mentioned in our response to **Reviewer 1, Q1)**, which provides statistical evidence supporting the presence of true modulation effects; **3)** we performed cell type proportion estimation analysis to address potential concerns that the modulation effect of meCpG might arise due to variations in methylation levels stemming from different cell type compositions within our samples (as discussed in our response to **Reviewer 3, Q2**). Taken together, we believed that the modulation effect of meCpG was now  well-supported by the presented evidence.

We sincerely hope the reviewer understands our current focus on introducing this innovative memo-eQTL mapping method, exploring its applications, and elucidating potential regulatory mechanisms. We wholeheartedly agree with the reviewer on the importance of experimental validation. It is worth noting that in our prior study [[6]](https://paperpile.com/c/v7YzmZ/OP4W), we successfully validated a memo-eQTL at the MYC locus, and the current study builds upon that foundation. Such validation experiments demand a considerable time investment, making them impractical even with the extended revision timeframe provided by the editor. While we are in the process of systematically validating the identified memo-eQTL, we believe that undertaking such a comprehensive effort falls beyond the scope of this specific study.

# Reviewer #3.

Zeng et al. introduced the concept of memo-eQTLs, a novel type of expression quantitative trait loci (eQTLs) regulated by DNA methylation of CTCF-binding sites. The authors revealed the existence of a previously overlooked group of eQTLs that would not have been detected without considering the statistical interaction between methylation and single nucleotide polymorphisms (SNPs). This submission emphasizes the intricate nature of eQTL identification and provides profound insights into the interplay between the genome and epigenome.

We thank the reviewer for recognizing the significance of our work and for providing valuable and insightful comments.

**Q1:** Applicability of the framework to other omics: DNA methylation variations can reflect differences in cell types or 3D genome structures. Can this analytical framework be adapted to investigate cell type-specific eQTLs or eQTLs specific to 3D looping?

We thank the reviewer for this insightful question. Given the inherent variability of DNA methylation levels across different cell types, we believe that, in theory, it is feasible to adapt our approach to explore meCpG modulated eQTLs in a cell type-specific manner. This is particularly the case when robust imputation or characterization of cell types is possible, such as through single-cell omics data. However, as the reviewer pointed out in Q2, one must be cautious when adapting this approach for bulk tissue samples composed of  diverse cell types with variable compositions.

In the current study, we have addressed potential concerns related to varying cell types through a comprehensive cell composition analysis, as elaborated in response to Q2. We have incorporated the findings into the **Results** section, as well as a thorough discussion on this topic in the **Discussion** section.

**Q2:** I guess much of the challenge of memo-QTL is from the limitation of data to tissues with variable cell type composition, leading to methylation change. Assuming proper control of cell types, how many of the memo-QTLs would have been identified as eQTLs?

We thank the reviewer for raising this insightful question. To address this, we estimated the cell type composition and variance within our samples. However, due to the limited overlap between the measured CpG sites in our samples and existing reference DNA methylation signatures, we encountered difficulties in employing well-established methods like EpiDISH [[7]](https://paperpile.com/c/v7YzmZ/H8F6) and MethylCIBERSORT [[8]](https://paperpile.com/c/v7YzmZ/DJAn) for cell composition estimation.

As an alternative, we employed EPIC [[9]](https://paperpile.com/c/v7YzmZ/BFLH) and utilized gene expression data to estimate the cell type proportions. Our analysis revealed that, on average, around 72% of the cells within our samples are non-immune cells (**Reviewer Figure 4A**). The detailed cell estimation results by both EPIC and xCell [[10]](https://paperpile.com/c/v7YzmZ/0YWs) have also been included in new **Supplementary Table 2**. Furthermore, our observations indicate that the major cell types (with median fraction > 0.2) tend to exhibit lower variation (with coefficient of variation (CV) < 0.3) (**Reviewer Figure 4B, C,** which has also been added as new **Fig. S2F, G**). Together, these results suggest that the variation in cell type composition within our sample is relatively small, which is in line with our expectation for healthy samples.

We agree with the reviewer that methylation variations might arise from the variable cell types and their compositions, especially in complex tissue samples like tumors. Although methods like ReFACTor [[11]](https://paperpile.com/c/v7YzmZ/DE6Z) and RUVm [[12]](https://paperpile.com/c/v7YzmZ/f0ye) have been proposed to adjust for the cell-type epigenome heterogeneity, it’s important to recognize that these algorithms vary quite substantially in their capacity to preserve genuine biological signals. No single method stands out as the most effective one across all contexts, as reviewed in reference [[13]](https://paperpile.com/c/v7YzmZ/nXSI). We have further explored this topic in the **Discussion** section.

**Reviewer Figure 4. Estimation of cell type composition and variance.  A)** Distribution of the fraction of six cell types estimated by EPIC. The relationship between the CV and Median of the cell fraction derived from EPIC **(B)** and xCell **(C)** estimations, respectively.

**Q3:** Independence assumption of methylation and SNP: The search for memo-QTLs began by focusing only on cases where all combinations of alleles and methylation states were present, automatically excluding cases where the SNP acted as a full meQTL. Is it possible for a SNP to simultaneously function as both an meQTL and a memo-QTL?

We thank the reviewer for this question. In our study, we didn’t explicitly exclude the possibility of the meQTL. In fact, out of the 1,063 memo-eQTLs, we identified 81 that were also recognized as meQTLs for 63 meCpG sites. This indicates that a single SNP could potentially influence both DNA methylation levels and gene expression simultaneously. The gene expression might be modulated and/or mediated by changes in DNA methylation at corresponding CTCF-binding sites. However, a comprehensive understanding of these simultaneous functions would require a more in-depth analysis and validation in subsequent studies. While our current study offers criticall insights into the regulation of gene expression due to the interplay between genetic and epigenetic factors, the exploration of such dual roles of meCpG would be an exciting avenue for future research endeavors. We have integrated this perspective into the **Results** section to acknowledge its importance.

# Reviewer Supplementary

To assess the performance of p-values-based method and FDR-based method in identifying significant memo-eQTLs, we devised an artificial dataset. This dataset employed the identical SNP and meCpG data for all tested SNP-CpG-Gene combinations from our current study, while the gene express data (y) was deliberately generated through randomization using the following formula:

𝑦 = β0 + β_1_𝑆𝑁𝑃 + β_2_𝑚𝑒𝐶𝑝𝐺 + β_3_𝑆𝑁𝑃 × 𝑚𝑒𝐶𝑝𝐺 + ε,

where different β values combination were randomly selected from the **Reviewer Supplementary Table 1** (which has been included in **Supplementary Table 2**)**,** and ~ N(0, 2) accounts of the normal distributed error term with the same sample size (N = 128). Subsequently, we conducted the memo-eQTL mapping for all SNP-CpG-Gene combinations using this artificial dataset. The real memo-eQTL was defined as when 30, and the predicted memo-eQTL was determined using both p-values-based and FDR-based methods. Lastly, both F1 scores (as defined below) and the count of the True Positives were examined to evaluate the performance of both methods.

$$F1= \frac{2*True Positive}{2*True Positive+False Positive+False Negative}$$

**Reviewer Supplementary Table 1**:  β values combinations used for simulation.

| **#** | **0** | **1** | **2** | **3** | 2 |
| --- | --- | --- | --- | --- | --- |
| 1 | 1 | 0.0 | 0.0 | 0.0 | 0.25 |
| 2 | 1 | 0.0 | 0.0 | 0.1 | 0.25 |
| 3 | 1 | 0.0 | 0.0 | 0.5 | 0.25 |
| 4 | 1 | 0.1 | 0.0 | 0.0 | 0.25 |
| 5 | 1 | 0.1 | 0.1 | 0.1 | 0.25 |
| 6 | 1 | 0.5 | 0.5 | 0.5 | 0.25 |
| 7 | 1 | 0.1 | 0.1 | -0.1 | 0.25 |
| 8 | 1 | 0.5 | 0.5 | -0.5 | 0.25 |
| 9 | 1 | 0.1 | 0.1 | 0.5 | 0.25 |
| 10 | 1 | 0.5 | 0.5 | 0.1 | 0.25 |
| 11 | 1 | 0.1 | 0.1 | -0.5 | 0.25 |
| 12 | 1 | 0.5 | 0.5 | -0.1 | 0.25 |

# Reference

[1. Li J, Xu C, Lee HJ, Ren S, Zi X, Zhang Z, et al. A genomic and epigenomic atlas of prostate cancer in Asian populations. Nature. 2020;580:93–9.](http://paperpile.com/b/v7YzmZ/7XCA)

[2. Giambartolomei C, Vukcevic D, Schadt EE, Franke L, Hingorani AD, Wallace C, et al. Bayesian test for colocalisation between pairs of genetic association studies using summary statistics. PLoS Genet. 2014;10:e1004383.](http://paperpile.com/b/v7YzmZ/zDaa)

[3. Gusev A, Ko A, Shi H, Bhatia G, Chung W, Penninx BWJH, et al. Integrative approaches for large-scale transcriptome-wide association studies. Nat Genet. 2016;48:245–52.](http://paperpile.com/b/v7YzmZ/u2aH)

[4. Rashkin SR, Graff RE, Kachuri L, Thai KK, Alexeeff SE, Blatchins MA, et al. Pan-cancer study detects genetic risk variants and shared genetic basis in two large cohorts. Nat Commun. 2020;11:4423.](http://paperpile.com/b/v7YzmZ/EeWp)

[5. Ahmed M, Mäkinen V-P, Mulugeta A, Shin J, Boyle T, Hyppönen E, et al. Considering hormone-sensitive cancers as a single disease in the UK biobank reveals shared aetiology. Commun Biol. 2022;5:614.](http://paperpile.com/b/v7YzmZ/rNyL)

[6. Ahmed M, Soares F, Xia J-H, Yang Y, Li J, Guo H, et al. CRISPRi screens reveal a DNA methylation-mediated 3D genome dependent causal mechanism in prostate cancer. Nat Commun. 2021;12:1781.](http://paperpile.com/b/v7YzmZ/OP4W)

[7. Zheng SC, Breeze CE, Beck S, Teschendorff AE. Identification of differentially methylated cell types in epigenome-wide association studies. Nat Methods. 2018;15:1059–66.](http://paperpile.com/b/v7YzmZ/H8F6)

[8. Chakravarthy A, Furness A, Joshi K, Ghorani E, Ford K, Ward MJ, et al. Pan-cancer deconvolution of tumour composition using DNA methylation. Nat Commun. 2018;9:3220.](http://paperpile.com/b/v7YzmZ/DJAn)

[9. Racle J, Gfeller D. EPIC: A Tool to Estimate the Proportions of Different Cell Types from Bulk Gene Expression Data. Methods Mol Biol. 2020;2120:233–48.](http://paperpile.com/b/v7YzmZ/BFLH)

[10. Aran D, Hu Z, Butte AJ. xCell: digitally portraying the tissue cellular heterogeneity landscape. Genome Biol. 2017;18:220.](http://paperpile.com/b/v7YzmZ/0YWs)

[11. Rahmani E, Zaitlen N, Baran Y, Eng C, Hu D, Galanter J, et al. Sparse PCA corrects for cell type heterogeneity in epigenome-wide association studies. Nat Methods. 2016;13:443–5.](http://paperpile.com/b/v7YzmZ/DE6Z)

[12. Maksimovic J, Gagnon-Bartsch JA, Speed TP, Oshlack A. Removing unwanted variation in a differential methylation analysis of Illumina HumanMethylation450 array data. Nucleic Acids Res. 2015;43:e106.](http://paperpile.com/b/v7YzmZ/f0ye)

[13. Teschendorff AE, Zheng SC. Cell-type deconvolution in epigenome-wide association studies: a review and recommendations. Epigenomics. 2017;9:757–68.](http://paperpile.com/b/v7YzmZ/nXSI)

**Second round of review**

**Reviewer #2:** The authors have conducted additional analyses to strengthen their study and have addressed my questions.

**Reviewer #3:** The authors responded to my prior review very well. I do not have further comments.
